# Improving Care for Deinstitutionalized People With Mental Disorders: Experiences of the Use of Knowledge Translation Tools

**DOI:** 10.3389/fpsyt.2021.575108

**Published:** 2021-04-26

**Authors:** Izabela Fulone, Jorge Otavio Maia Barreto, Silvio Barberato-Filho, Cristiane de Cássia Bergamaschi, Marcus Tolentino Silva, Luciane Cruz Lopes

**Affiliations:** ^1^Pharmaceutical Sciences Graduate Course, University of Sorocaba, Sorocaba, Brazil; ^2^Fiocruz School of Government, Fiocruz Brasília, Oswaldo Cruz Foundation, Brasília, Brazil

**Keywords:** evidence-informed policy, knowledge translation, deinstitutionalization, mental health, community mental health services

## Abstract

**Background:** The deinstitutionalization process is complex, long-term and many countries fail to achieve progress and consolidation. Informing decision-makers about appropriate strategies and changes in mental health policies can be a key factor for it. This study aimed to develop an evidence brief to summarize the best available evidence to improve care for deinstitutionalized patients with severe mental disorders in the community.

**Methods:** We used the SUPPORT (Supporting Policy Relevant Reviews and Trials) tools to elaborate the evidence brief and to organize a policy dialogue with 24 stakeholders. A systematic search was performed in 10 electronic databases and the methodological quality of systematic reviews (SRs) was assessed by AMSTAR 2.

**Results:** Fifteen SRs were included (comprising 378 studies and 69,736 participants), of varying methodological quality (3 high-quality SRs, 2 moderate-quality SRs, 7 low-quality SRs, 3 critically low SRs). Six strategies were identified: (i). Psychoeducation; (ii). Anti-stigma programs, (iii). Intensive case management; (iv). Community mental health teams; (v). Assisted living; and (vi). Interventions for acute psychiatric episodes. They were associated with improvements on a global status, satisfaction with the service, reduction on relapse, and hospitalization. Challenges to implementation of any of them included: stigma, the shortage of specialized human resources, limited political and budgetary support.

**Conclusions:** These strategies could guide future actions and policymaking to improve mental health outcomes.

## Background

Deinstitutionalization is the procedure of shifting the care and support from long-stay psychiatric hospitals to community mental health services for patients diagnosed with severe mental disorders (1). This procedure works in two ways. The first concentrates on reducing the population size of mental institutions. The second emphasizes reforming psychiatric care and developing special services to reduce dependence, isolation and other behaviors that make it difficult for patients to adjust to life outside of care (2).

Deinstitutionalization emerged in the post-World War II period in the 1950s in the US and the UK due to several factors, such as poor and inhumane living conditions, human rights violations, harmful treatment practices, the introduction of more effective psychotropic drugs and the high cost of mental hospitals (3). Although many countries have advanced and reached positive levels in this process, such as USA, England, Italy, Germany and UK, others are still starting the process and are facing many problems (4). Many challenges remain in low-and middle-income countries, Eastern Europe, and Eastern and Southeastern Asian countries (5, 6).

This complex process entails ensuring access to and developing special alternative community services for the care of the physical and mental health of the mentally ill, non-institutionalized population, with the aim to improve quality of life, ensure citizenship and promote social inclusion (2, 7).

Many countries fail because they close institutions without careful planning and without implementing community (8). Failures to establish basic infrastructure, to diversity and to integrate the mental health services are the most common (9). This fact can have serious effects such as homelessness, marginalization, and “reinstitutionalization” or “transinstitutionalization” into prisons or asylums as well as worsening psychiatric conditions and crowding emergency department (10).

Informing decision makers about positive strategies and appropriate changes in mental health policies could be a key factor for mental healthcare development (10, 11). Considering Brazil, as a case scenario, this study aimed to identify effective strategies to improve care for deinstitutionalized patients with mental disorders in the community, through the use of knowledge translation tools.

## Method

**Figure 1:**
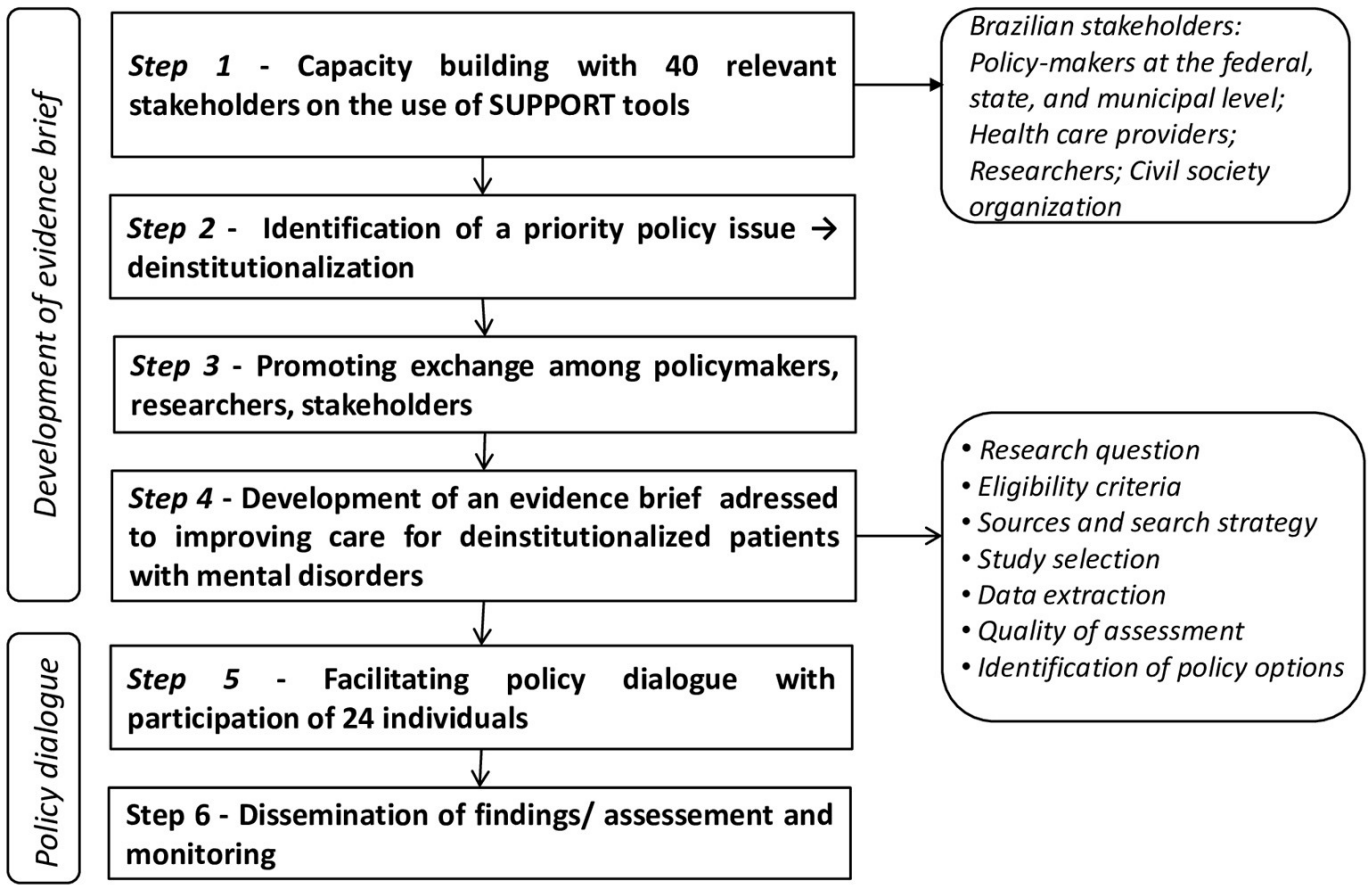
Steps used on elaboration of evidence brief.

The SUPporting POlicy relevant Reviews and Trials (SUPPORT) tools (12, 13) were used to guide the process as methods to obtain evidence to inform health policymaking and to develop an evidence brief and to organize the policy dialogue, Figure 1.

### Eligibility Criteria of the Studies

#### Inclusion Criteria

##### Participants

Patients 18 years of age and older suffering from severe and persistent non-affective mental disorders (schizophrenia and schizophreniform, schizoaffective or schizotypal disorders or multiple diagnoses) who were or not institutionalized in psychiatric hospitals.

##### Interventions

Strategies for outpatient follow-up and care in the community.

##### Comparator

Comparison with usual/standard care, other strategies for outpatient follow-up and care in the community or nothing.

##### Outcome

Compliance with medication, relapse, satisfaction with the service, internalized stigma reduction, reduction in stigmatizing attitudes, hospitalization, contacts with mental health services, improve of the global and mental state, social rehabilitation status, quality of life, death by suicide, stability, equity, harms and costs.

##### Timing

Any duration of follow-up.

##### Studies Design

Systematic reviews (SR), overview of SRs and economic assessment studies. We selected these study designs because they are at the top of the hierarchy of evidence pyramid.

#### Exclusion Criteria

We excluded articles that evaluated only the clinical outcomes related to psychiatric patients without providing information regarding management strategies, actions and/or methodologies for the process of monitoring deinstitutionalized patients, as well as outdated SRs whose topics have been addressed in updated SRs. Studies that reported results only for patients with mild mental disorders or with dementia or intellectual disorders or substance abuse or for people with mental disorders who were already living on the street (homeless) were excluded.

### Sources of Information and Search Strategy

The electronic search of eligible studies was performed in the following databases until 13 January 2020: Virtual Health Library, Cochrane Library, PubMed, Health Evidence, Rx for Change, Cumulative Index to Nursing and Allied Health Literature, Excerpta Medica Database, American Psychological Association, Epistemonikos, Latin American & Caribbean Health Sciences Literature.

We also screened the reference lists of secondary studies and manually searched for references in journals and databases. We did not apply any limits on language or date of publication. The search strategy in Medline (Ovid) is presented in Supplementary Data Sheet 1. We adapted it to each database.

### Study Selection Process and Data Extraction

Two review authors (IF, CB) independently screened the titles and abstracts for inclusion. Then, the full texts of potentially relevant references were retrieved, and two review authors (IF, CB) independently assessed the full-text articles for inclusion and extracted all relevant data. Any disagreements were resolved by a third review author (LCL). Data extracted included the following: author, year, type and number of primary studies included, year range of the primary studies, setting of included studies, total number of subjects, type of intervention and of comparator, type of outcome measure and main outcomes.

We also checked barriers and facilitators general for implement strategies for outpatient follow-up and care in the community and their inequities. To verify inequities for implement health policies, we used the PROGRESS (place of residence, race/ethnicity/culture/language, occupation, gender/sex, religion, education, socioeconomic status, and social capital) framework to ensure considerations for health equity (14).

### Quality Assessment of Systematic Reviews

The quality of the SRs was assessed using the updated “A Measurement tool for Assessing the Methodological Quality of Systematic Reviews” (AMSTAR 2) (15).

AMSTAR 2 considers seven critical domains (items 2, 4, 7, 9, 11, 13, and 15) to rate the overall confidence in the findings of each SR.

### Organization of Policy Dialogue

We used SUPPORT tools to guide the organization of the policy dialogue and to discuss the evidence brief and validate it with relevant stakeholders involved in the problem. From meetings with policy-makers researchers and stakeholders, key informants were identified and invited to participate of the policy dialogue. Twenty-four individuals participated of policy dialogue (5 of them were policymakers, 11 health care providers, 6 researches, 1 from civil society organization and 1 representant from public defense). The process, outcomes and lessons learnt during this dialogue were showed in details in elsewhere (16).

A preliminary version of the evidence brief was pre-circulated among participants and the strategies and key implementation considerations were discussed exhaustibly during the policy dialogue. After, the evidence brief was aligned and updated according to the deliberations and outputs produced.

## Results

**Figure 2:**
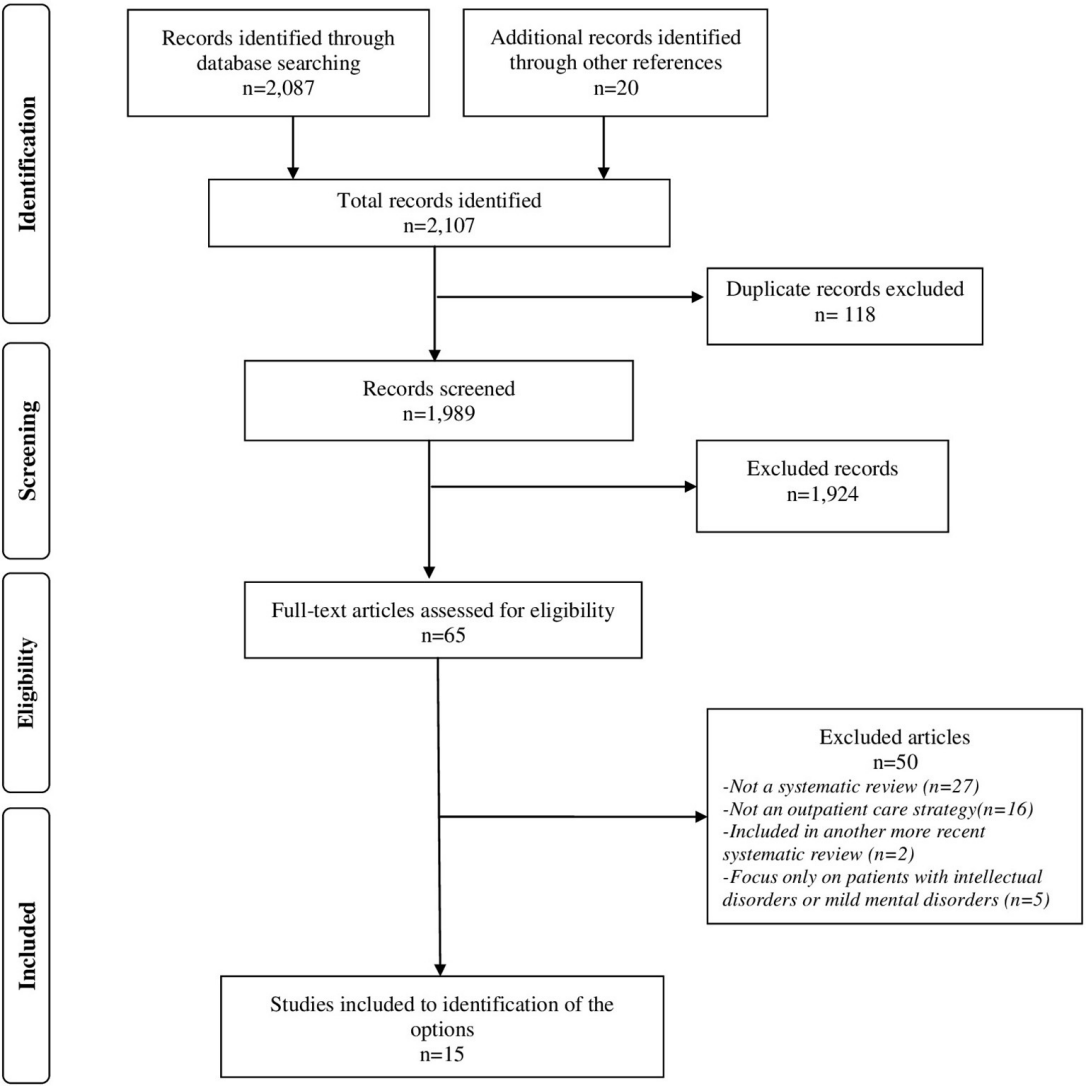
Flow diagram for study selection.

Overall, 2,107 references were retrieved. Sixty-five studies were selected and examined in detail; fifteen SRs met the scope of this evidence brief and were selected to develop the policy strategies (see detailed results reported in Supplementary Table 1), while fifty studies were excluded (see Supplementary Table 2). A flow diagram illustrates the inclusion process, Figure 2.

From the 15 SRs, we identified six strategies to improve care for deinstitutionalized patients: (i). psychoeducation; (ii). anti-stigma programmes; (iii). intensive case management; (iv). community mental health teams; (v). assisted living; and (vi). interventions for acute psychiatric episodes. The main characteristics of the included reviews are summarized in Table 1.

**Table 1:** Characteristics of the included systematic reviews.

**Author, Year**	**Number of studies included**	**Year range of the studies**	**Total number of subjects**	**Main outcomes**	**Quality of assessment (AMSTAR 2)**
**Strategy 1: psychoeducation**					
Pilling, 2002**	18	1978–1997	1,467	- Relapse - Readmission - Death (suicide) - Burden, expressed emotion - Medication compliance	critically low
Lincoln, 2007**	18	1982–2005	1,534	- Relapse/rehospitalization - Symptoms - Knowledge - Functional outcome - Medication adherence	low
Xia, 2011**	44	1988–2009	5,142	- Compliance with medication and Follow- up - Relapse - Satisfaction with the service	moderate
Zhao, 2015*	20	1988–2009	2,337	- Compliance with medication and follow- up - Relapse	high
**Strategy 2: anti-stigma programs**					
Tsang, 2016**	14	2007–2015	1,131	- Reduction in internalized stigma	critically low
Wood, 2016**	12	2002–2016	714	- Improvement in internalized stigma	low
Xu, 2017**	17	2011–2015	2,373	- Effects on perceived/ experienced/anticipated stigma - Effects on self-prejudice - Effects on stigma coping	critically low
Morgan, 2018**	62	2001–2017	9,002	- Reductions in stigmatizing attitudes - Desire for social distance	low
**Strategy 3: intensive case management**					
Burns, 2007**	29	1988–2005	1,996	- Days of hospitalization	low
Dieterich, 2017**	40	1985–2005	7,524	- Hospitalization - Improve of global state - Reducing death by suicide - Social functioning (on unemployment)	high
**Strategy 4: community mental health teams**					
Malone, 2017**	3	1992–1998	587	- Death (suicide /suspicious circumstances) - Hospitalization - Satisfaction with the service - Social functioning	moderate
**Strategy 5: assisted living**					
Leff, 2009*	44	1983–2006	13,436	- Housing stability - Reduction in psychiatric symptoms - Reduction in hospitalization - Reduction in alcohol abuse or drug abuse - Increased employment - Increased satisfaction	low
McPherson, 2018*	28^£^	1990–2017	6,516^#^	- Housing stability - Hospitalization - Symptoms of mental illness - Social functioning	low
**Strategy 6: interventions for acute psychiatric episodes**					
Murphy, 2015**	8	1964–2010	1,144	- Hospitalization - Improve mental state and global state - Satisfaction with the care - Quality of life - Burden family	high
Wheeler, 2015*	21^&^	1993–2011	14,833^##^	- Hospital admissions - Characteristic of service	low

*Systematic review without meta-analysis; **Systematic review with meta-analysis; ^£^Studies included in deinstitutionalization subgroup; ^&^Studies included in the quantitative analysis; ^#^One study did not declare total n; ^##^Seven studies did not declare total n.

**Figure 3:**
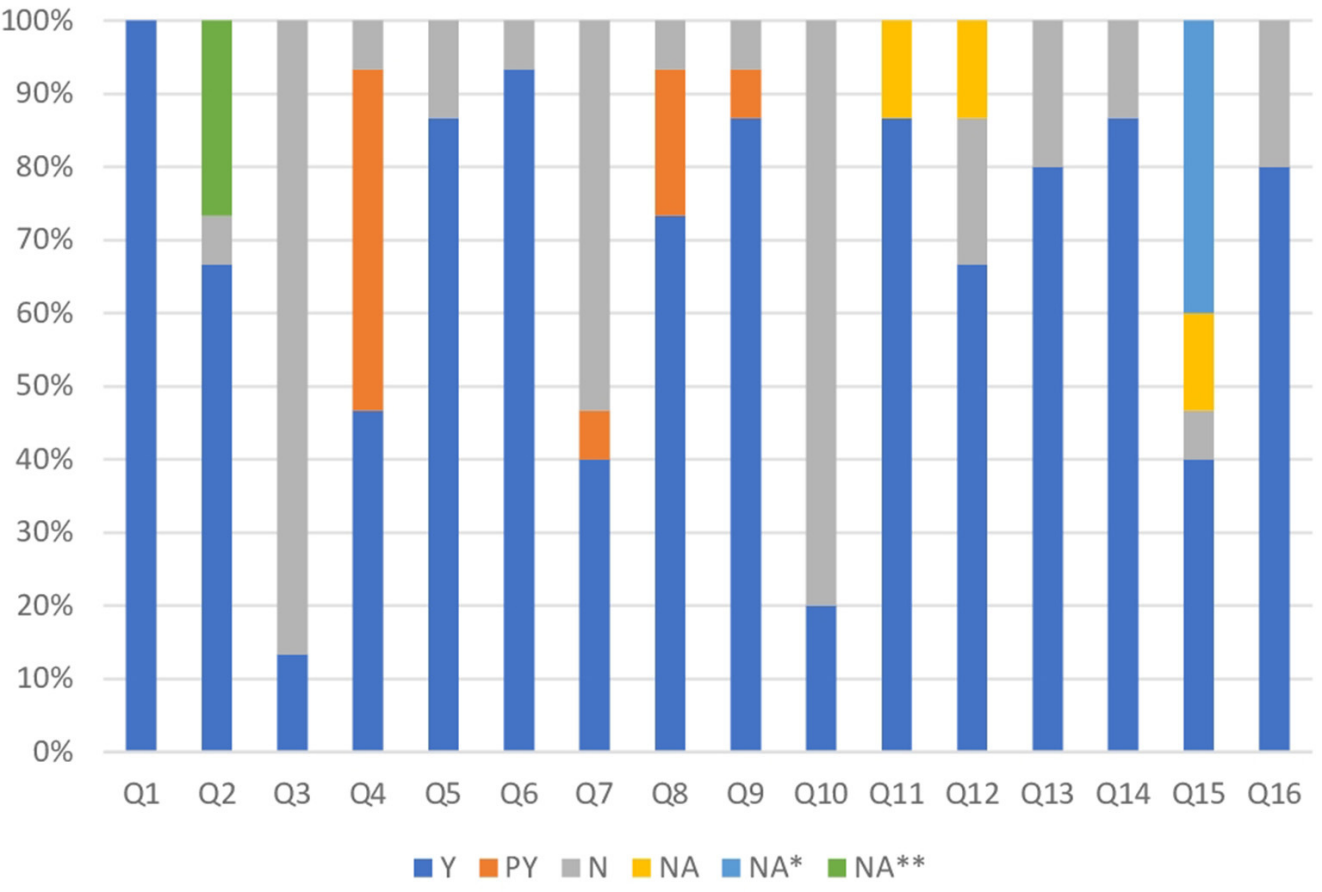
Comparison of quality assessment of included reviews using AMSTAR 2 criteria. Y, yes; PY, partial yes; N, no; NA, not applicable; NA*, not applicable because there were 10 or fewer studies per outcome; NA**, the systematic review protocol records base PROSPERO was available virtually in February 2011.

As already noted in the Table 1, three (20.0%) SRs were considered high in quality, two (13.3%) moderate, seven (46.6%) low, and three (20.0%) critically low. Weaknesses were found in items 3, 4, 7, and 10. Thirteen (86.6%) of the included SRs failed to provide justification for their selection of study designs (item 3). A comprehensive literature search strategy was revealed in seven (46.6%) of the SRs, but the remaining studies failed to do so because they did not show a justification for language restrictions or did not search for gray literature (item 4, critical domain). Eight (53.3%) of them did not provide a list of excluded studies that were read in full-text form or report reasons for their exclusion (item 7, critical domain). Twelve (80.0%) of them did not report on the sources of funding for the studies included (item 10), Figure 3. AMSTAR results are provided in Supplementary Table 3.

No potential harm or cost-effectiveness was pointed out in the SRs included. There are few cost data, and no conclusions regarding cost-effectiveness can be drawn. The findings of each strategy showed in the SRs are summarized as follow.

### Strategy 1: Psychoeducation

Four SRs (17–20) addressed the effectiveness of psycho-educational programmes as a means of improving care for severely mentally ill people.

Psychoeducation involves any group or individual programme with a combination of motivational, educational and behavioral techniques focused on knowledge and understanding of the disease, symptoms, treatment, prognosis and rehabilitation, and it should be directed to the patient, caregivers and family members (19).

When this intervention is addressed to patients, it promotes greater adherence to treatment in the short, medium and long term, lower relapse rates in medium and long term and greater satisfaction with the service (19, 20).

Nevertheless, psychoeducation with families (*n* = 18 studies) showed more effective in reducing relapse/rehospitalization (follow-up 7–12 months) than psychoeducation without families. The effect size for knowledge was small and was no significant effect on symptoms change, functioning and medication adherence. At longer follow-up (>12 months), the results on relapse/rehospitalization also failed (18).

Aside from that, educational interventions offered to caregivers or families in comparison to all other treatments, standard care or other types of active treatments have shown benefits over relapse in the first 12 months, but this effect was not sustainable between 1–2 years. The single-family interventions demonstrated greater effectiveness over group family treatments to prevent readmissions in the long term (1–2 years) and to reduce the burden (17). The both SRs (17, 18) suggest the additional effort to integrating families and to offering psychoeducational interventions for longer periods.

### Strategy 2: Anti-stigma Programs

Four SRs were addressed the reduction of stigma (21–24). This option came after discussions about the Psychoeducation strategy with stakeholders. As some studies showed specific results, directing psychoeducation to reduce stigma, the participants suggested separating psychoeducation strategies that focused directly on reducing stigma and those related to the education of family members or patients to learn about the disease

Many interventions have been developed to reduce the negative impact, the discrimination and misconceptions around the public stigma and of internalized stigma toward people with severe and persistent mental disorders (24). The main approaches include psychoeducation, combined or not with other components, such as cognitive behavioral therapy, social skills training or group discussion elements (21–23).

Some SRs (21, 23) showed that psychoeducation was effective to reduce the internalized stigma and the self-prejudice. On the other hand, a SR (*n* = 12 studies) involving psychoeducation and/or other of psychosocial interventions (cognitive behavior therapy, social skills training, photovoice) did not found significant changes in internalized stigma at the end of the therapy or at follow up to 4 months (22).

To reduce the public stigma toward people with severe mental illness, education interventions, mixed or not with contact interventions, showed immediate positive effects. At the end of the treatment, stigmatizing attitudes and desire for social distance were reduced, but at follow up 6 months, the benefits were not sustainable (24).

### Strategy 3: Intensive Case Management

Two SRs (25, 26) highlighted the effectiveness of the practice of intensive case management. This strategy is characterized by an integrated model of health care delivery and follow-up that aims to provide systematic, flexible and coordinated mental health services according to the health and social care needs of people with severe mental illness (26).

This intervention model decreased the number of days of hospitalization, increased the retention in care, improved global state and promoted greater patient satisfaction (26). Nevertheless, other studies have shown a reduction in the hospitalization rate only for patients at high risk of hospital admission, who tend to use more of these services than patients who already have low hospitalization rates (25).

### Strategy 4: Community Mental Health Teams

One SR (27) addressed the effectiveness of community mental health teams. A community mental health team is a multidisciplinary team composed of specialists in mental health, who should lead and be responsible for providing expert assessment, treatment and care to the population of a given area in the community (27). These team is different of other services including crisis intervention (24 h service) or assertive community treatment (restricted caseloads).

It can be a way of integrating mental health into primary care. In addition, having greater contact with patients and families makes it possible to detect and intervene earlier in some serious symptoms or other diagnoses (28). Community mental health team follow-up promotes greater patient satisfaction with the service, lower hospital admission rate than standard care (without community mental health teams) and improvement of social functioning including police contacts. Although the evidence is still insufficient, follow-up performed by such teams tends to reduce the number of suicides (27).

### Strategy 5: Assisted Living

Two SRs (29, 30) addressed the benefits of community housing models for deinstitutionalized persons with severe mental illness. Post-deinstitutionalization, assisted living emerged due to the housing needs for former patients of large psychiatric hospitals who had been resettled in the community. Housing models vary in terms of their physical structure, staffing arrangements, levels of support, recovery focus, discharge and move-on policies (30).

Patients living in residential care and treatment model housing have shown greater stability, reduction in hospitalization and in psychiatric symptoms (29).

### Strategy 6: Interventions for Acute Psychiatric Episodes

Two SRs (31, 32) addressed the effectiveness of models for interventions for acute psychiatric episodes. Interventions in acute psychiatric episodes should provide rapid assessment and intensive treatment for a brief period through a multidisciplinary team specialized in crisis situations either in a community setting or in the patient's own home. Such interventions represent a viable alternative that is less stigmatized than standard hospitalization (31).

Despite the results on hospitalization, improvement in mental and global status, and quality of life remains inconclusive, crisis interventions promote greater satisfaction with treatment and less burden on family compared to standard care received in a hospital (31). In other SR (*n* = 21 studies), it was not feasible to summarize the data due to the variety of design of included studies, but suggest to reduce hospitalizations and highlight some key components that should be available: 24-h service provision, including psychiatrists, high-quality staff training and integration with other local mental health services (32).

### Implementation Barriers and Inequities

The planning and implementation in mental health policies should consider the characteristics of the option itself, the outer setting (social, political and economic context), the inner setting (structural characteristics, relationships) and the characteristics of the individuals involved (knowledge, skills) (33). Some of these factors can represent barriers that are likely to be encountered at the political, professional, patient and societal levels. Some common barriers in mental health are showed in Supplementary Table 4.

Stigma, discrimination, cultural beliefs and negative societal responses to people with mental illness are recognized as one of the largest barriers in the mental health area (34), remaining strong in society, in patients and among health professionals. It is necessary to raise public awareness and promote education campaigns (35).

Although the staff composition of health professionals varies by setting, population needs, the type of health system and the availability of financial resources, the shortage of appropriate human resources for mental health, particularly in low-middle-income countries, is recognized as a global concern (36, 37). Establishing effective training programmes, clear documentation practices and supervision about quality of services are strategies recommended (36, 38).

Low political priority, insufficiency resource, knowledge-action gap in policy implementation and lack of partnership formation with other sectors are important obstacles that needs to be overcome (33, 37, 39).

Cooperation from all levels of government to implement and review mental health policy, configuration of proactive partnerships and the adoption of scientific implementation frameworks are facilitators to improve the care practice (11, 33, 37).

Half the strategies implemented delivered effective outcomes in the replicate sites due contextual differences, inequalities and the unpredictable behavior of the system (39). Some groups or places may be potentially disadvantaged or under different conditions, which obscures the effectiveness of an option.

People with low socioeconomic conditions, with physical disability or frailty, and who lives in rural area were considered to be potentially disadvantaged. Poor rural people have few or no local treatment options and their access in the city is expensive. They are also less likely to achieve long term follow-up (9). Strategies to overcome these iniquities include integration to primary care, subsidy for treatment and facility transportation in emergency cases (35). WHO recommends the integration and strength of primary healthcare to mental health services in order to decrease the global gap in mental health (40).

### Main Contributions Obtained in the Policy Dialogue

- Psychoeducation was the strategy that received the most endorsement from all participants of the policy dialogue;
- Anti-stigma programs were added as one of the post-dialogue suggestions;
- Intensive case management and assisted living were recognized as one the main axes of deinstitutionalization, but there is the need to improve their structure and organization;
- Despite not have in Brazil, community mental health teams were considered promising strategy.
- Interventions for acute psychiatric episodes were realigned post-dialogue and were the most discussed. The prominent discussion emerged around ensuring a brief and intensive treatment, and defending the end of hospitalization for long periods.
- The deliberations related to the implementation barriers focused mainly on the stigma, lack of funding and political will. Participants emphasized that the stigma of being labeled as a deinstitutionalized patient needs to change and can no longer be considered as an unpredictable, dangerous individual, unable to live in the community. Perhaps, overcoming stigma is the biggest challenge.

## Discussion

The available evidence from 15 SRs covered six different types of strategies that can lead to meaningful improvements in care for deinstitutionalized people with mental disorders and their health outcomes. They can complement each other, but not necessarily have to be employed together. The outcomes, estimates of effects, and the quality of SRs varied. The paucity of studies and conflicting evidence has been observed in some strategies. The deliberations obtained in the policy dialogue contributed to align the strategies, to improve the evidence brief and validate it.

There was extensive evidence for the positive effects of the psychoeducation (strategy 1), but the true benefits and cost-effectiveness in the short and long-term still are uncertain (19, 20), as well as whether it is better to apply group delivery rather than individually, or only with patients or with the family (17, 18). Similarly, the wide variety of combined strategies in the anti-stigma programs (strategy 2) also showed conflicting results and it was unfeasible to determine whether there is any recommendation on which strategy or duration is most effective (21–24).

The lack of fidelity to maintain and apply key components in the structure and organization aspect of an original model as Intensive case management (strategy 3) could explain the variation in some outcomes (e.g., hospitalization) between studies and the level of effectiveness (25, 26). Not all studies measured fidelity adequately to the original strategy.

Despite the number of primary studies existing in some strategies such as Assisted living (strategy 5) and Interventions for acute psychiatric episodes (strategy 6), the wide variety of instruments used to measure clinical and non-clinical results, the heterogeneity of the retrieved studies designs and the definitional inconsistency makes it impossible to combine some data, which reduces the power of conclusion and the degree of evidence confidence. Two SRs were unable to summarize the data due to the heterogeneity of the recovered study designs (30, 32). The lack of consistency in the definition of active components or terminology used in the published literature about assisted living models (strategy 5) has limited the evidence on which model is most effective and safe (30).

Whilst we found more studies within of some strategies, there was the strategy 3, Community mental health teams, with only a single SR, which included three trials (27). Some evidence is scarce and much more robust studies are needed.

We were unable to investigate the potential for harms associated with these strategies that might influence benefits, because any SR reported adverse events. Cost-effectiveness and consequences of implementing any of these strategies as a routine service was not assessed. Much more studies should be undertaken in this area to explore the costs, to measure the health economic outcomes and the harms of the strategies, in order to make them more attractive for managers and policymakers.

Considerations about the implementation barriers of any of the strategies are complex should be interpreted with caution. Barriers and facilities have dynamic nature, change over time and may be more or less affected according the extern context (41).

There is a real need to support evidence-based policy making. Combining research evidence with views, experiences and tacit knowledge from relevant stakeholders is a promising strategy. Policy dialogue strengthened interactions with policy makers, stakeholders and research and raised awareness of the importance of applying evidence to policies. Positive lessons have occurred in other countries (42, 43) and need to be disseminated worldwide, especially in low- and middle-income countries.

## Strengths and Limitations

This study evaluated a wide range of interventions and summarized in a single document the best evidence available to improve the care of patients with deinstitutionalized mental disorders in the community, including some implementation barriers, facilitators, and equity considerations. This policy brief is not restricted to only one audience, can reach mental health professionals, researchers, and policymakers and likely easier to be understood. It is also one of the few studies that reported experiences of use of knowledge translation tools combining development of an evidence brief and organization of policy dialogue in a middle-income country and addressed the issue of deinstitutionalization.

The majority of the SRs focused on high-income countries (United States of America, the United Kingdom, Canada), which revealed a gap in low-income countries. Considering Brazil as a case scenario, we could verify that although it has implemented several of these strategies, we did not find any SR including assessment of them in the Brazilian setting.

Some strategies were based on studies with low quality of evidence, limiting confidence in their findings. Some outcomes are under-researched such as cost, cost-benefit, harms, implementation barriers and equity.

Further studies should be conducted in low-middle-income countries because several factors are very different and, in some cases, deficient. There is a need to know the challenges they may face and whether the results are generalizable for these contexts.

More rigorous methods are needed to improve the validity of SRs, to provide high-quality evidence, and to increase the applicability of the findings by decision-makers. In addition, much more effects need to be explored and well-reported. Emphasis should be given to underreported outcomes, which involve patient outcomes and the advance of public health, harms, costs and inequities.

## Conclusions

This evidence brief showed six strategies based on the best evidence available and considering the strengths and weaknesses of each to improve care for deinstitutionalized people with severe mental disorders. The intention is not to advocate specific strategies or to for close discussion but to inform and to promote deliberations among policymakers and stakeholders with regard to the preferred strategies and their planning of implementation according to needs, financial resources, feasibility, the local reality and engagement among key actors. Thus, far, there is no consensus regarding which key components and implementation strategies are essential for successful mental health care service in the community.

## Data Availability Statement

The data generated and analyzed during the current study are not publicly available but are available from the corresponding author on reasonable request.

## Author Contributions

LL conceptualized the study. IF, JB, and LL designed the study. IF, LL, SB-F, CB, and MS participated in the study search strategy process. IF and CB participated in the study selection process and extraction of data. IF and LL assessed the quality of studies and drafted the manuscript. All authors contributed to and have approved the final manuscript.

## Conflict of Interest

The authors declare that the research was conducted in the absence of any commercial or financial relationships that could be construed as a potential conflict of interest.
