# Supplementary material for: Improving Care for Deinstitutionalized People With Mental Disorders: Experiences of the Use of Knowledge Translation Tools
Source: Front Psychiatry. 2021 Apr 26;12:575108. doi: 10.3389/fpsyt.2021.575108 (PMC8109270; doi:10.3389/fpsyt.2021.575108)
Supplement: Supplementary file 1 [file Table_1.PDF]

SUPPLEMENTARY MATERIAL

Table S1 – Included studies

Table S.1: Included studies (for strategy 1 – Psychoeducation).

Author, Year	Type and number of primary studies	Number of participants	Countries (studies)	Intervention	Comparator	Type of outcomes measure	Primary outcomes	AMSTAR 2
**STRATEGY 1 – PSYCHOEDUCATION**								
Pilling, 2002	randomised controlled trials (18)	1,467	not reported	family interventions:<br>i. psychoeducational intervention;<br>problem solving crisis management<br>work; or, intervention with the identified patient;<br>ii. cognitive behavior therapy.	standard care or active care	at least 6 weeks	**Family interventions versus standard care:**<br><br>**-relapse in first 12 months** (OR: 0.37, 95% CI 0.23 to 0.60; NNT=6);<br><br>**-relapse in follow-up 4-15 months after the end of the treatment, single family treatment** (OR: 0.70, 95% CI 0.7 to 1.76);<br><br>**-readmissions in first 12 months:** (OR: 0.43, 95% CI 0.08 to 2.28);<br><br>**-readmissions in the first 2 years, single family interventions** (RR: 0.39, 95% CI 0.11 to 1.34, NNT=9);<br><br>**-readmissions in follow-up up to 2 years after** (OR: 1.08, 95% CI 0.64 to 1.83, NNT= -18);<br><br>**-suicide** (OR:0.88, 95% CI 0.33 to 2.32);<br><br>**-burden** (WMD: -0.14, 95% CI -	Critically low

							0.76 to 0.47);<br><br>**-burden, single family treatment** (WMD: -0.42, 95% CI -0.88 to 0.03);<br><br>**-expressed emotion** (RR: 0.90, 95% CI 0.48 to 1.72, p= 0.38);<br><br>**-compliance with medication** (RR: 0.63, 95% CI 0.40 to 1.01, p= 0.65);<br><br>**Family interventions versus all other treatments:**<br><br>**-relapse in first 12 months** (OR: 0.52, 95% CI 0.31 to 0.89)<br><br>**-relapse in first 2 years, single family treatment** (OR: 0.57, 95% CI 0.18 to 1.82)<br><br>**-readmissions in first 12 months** (OR: 0.38, 95% CI 0.10 to 1.40)<br><br>**-readmissions in first 12 months, single family intervention** (OR: 0.22, 95% CI 0.09 to 0.51)<br><br>**-readmissions in first 2 years** (OR: 0.47, 95% 0.23 to 0.96)<br><br>**-compliance with medication** (OR: 0.63, 95% CI 0.40 to 1.01)<br><br>**Family interventions versus active treatments:**<br>**-relapse in first 12 months** (OR: 1.67, 95% CI 0.71 to 0.31)	

Lincoln, 2007	randomised controlled trials (18)	1,534	Great Britain (5); China (4), Germany and Switzerland (3),<br>Greece (1), Scandinavia (2), USA and Canada (3).	Psychoeducation with a focus on conveying relevant information about the disorder<br>and its treatment while promoting better coping.	non-active group (waiting-list, treatment usual, or a non-specific intervention without proven effectiveness, e.g. problem solving; supportive treatment, leisure time groups)	follow-up 6 months;<br>7–12 months,<br>>12 months	**Post-assessment:**<br><br>**-relapse/ rehospitalization** (d= 0.53, 95% CI 0.12- 0.95,<br>p= 0.01);<br><br>**-symptoms** (d=0.29, 95% CI −0.13–0.70, p= 0.08);<br><br>**-functional outcome** (d= −0.03, 95% CI −0.84–0.78, p= 0.97);<br><br>**-knowledge** (d= 0.48, 95% CI 0.12–0.83, p= 0.00);<br><br>**-medication adherence** (d=−0.25, 95% C −1.25–0.75,<br>p= 0.31);<br><br>**Follow-up ≤ 6 months:**<br><br>**-relapse/ rehospitalization** (d= 0.35, 95% CI 0.14–0.55, p= 0.00);<br><br>**Follow-up 7–12 months:**<br><br>**-relapse/ rehospitalization** (d= 0.48, 95% CI 0.15–0.82, p=0.00);<br><br>- **symptoms** (d=0.19, 95% CI −0.16–0.55, p= 0.14);<br><br>- **functional outcome** (d= −0.19, 95% CI −0.59–0.97, p= 0.32);<br><br>**Follow-up > 12 months:**<br>**-relapse/ rehospitalization**<br>(d=0.21, 95% CI −0.07–0.49,<br>p= 0.07)	Low

							**Psychoeducation with family:**<br><br>**-symptoms at post-assessment** (d=0.33, 95% CI −0.26–0.93, p= 0.14)<br><br>**-relapse/rehospitalizations at 7–12 month-follow-up** (d= 0.48, 95% CI 0.10–0.85, p= 0 .00)<br><br>**Psychoeducation without family:**<br><br>**-symptoms at post-assessment** (d= 0.24, 95% CI −0.39–0.86, p= 0 .23)<br><br>**-relapse/rehospitalizations at 7–12 month-follow-up** (d=0.18, 95% CI −0.47–0.82)	
Xia, 2011	randomized controlled trials (44)	5,142	China (32)<br>France (1)<br>USA (3)<br>Canada (1),<br>Germany (2)<br>UK (3)<br>Demark (1)<br>Malaysia (1)	psychoeducation (didactic interventions or patient teaching involving individuals or groups)	standard care (normal level of psychiatric care provided in the area where the trial was carried out).	short term: up to 12 weeks), medium term: 13-52 weeks, long term: over 52 weeks	**- compliance with medication in short term** (RR: 0.52, 95% CI 0.40 to 0.67; I^2=1%); **medium term** (0.36; 95% CI 0.27 to 0.49; I^2= 0%); **long term** (RR: 0.48, 95% CI 0.31 to 0.75; I^2= 78%);<br><br>**-compliance with follow up in medium term** (RR: 1.00, 95% CI 0.79 to 1.26; I^2= 30%), **long term by 2 years** (RR: 0.87, 95% CI 0.62 to 1.10; I^2= 0%), **long term by 5 years or more** (RR: 0.77, 95% CI 0.48 to 1.23; I^2= 0%)<br><br>**-relapse for any reason in medium term** (RR: 0.70, 95%CI 0.61 to 0.81; I^2= 59%); **long term** (RR: 0.73, 95% CI 0.62 to 0.85; I^2= 31%);	Moderate

							-**satisfaction with the service** (RR: 0.24, 95% CI 0.12 to 0.50);	
Zhao, 2015	randomised controlled trials (20)	2,337	China (10)<br>Germany (3)<br>UK (2)<br>Italy (1)<br>Malaysia (1)<br>Pakistan (1)<br>Denmark (1)<br>Jamaica (1)	brief psychoeducation (didactic interventions or patient teaching) with 10 or less sessions;	standard care (normal level of psychiatric care provided in the area where the trial was carried out)	short term: up to 12 weeks, medium term: 13-52 weeks, long term: over 52 weeks	**- compliance with medication in short term** (RR: 0.63, CI 0.41 to 0.96); **medium term** (RR: 0.17, 95% CI 0.05 to 0.54);<br><br>**- compliance with follow-up in short term** (RR: 1.00, CI 0.24 to 4.18), **medium term** (RR: 0.74, 95% CI 0.50 to 1.09), **long term** (RR: 1.19, 95% CI 0.83 to 1.72)<br><br>**- relapse in medium term (**RR: 0.70, 95% CI 0.52 to 0.93)	High

Table S.1 (continued): Included reviews (for strategy 2 – Anti-stigma programs).

Author, year	Type and number of primary studies	Number of participants	Countries (studies)	Intervention	Comparator	Type of outcome measure	Primary outcomes	AMSTAR 2
**STRATEGY 2 –ANTI-STIGMA PROGRAMS**								
Tsang, 2016	randomised controlled trials (7); controlled clinical trials (3); uncontrolled studies without a control group (4);	1,131	US (5) Canada (2) Israel (1); Japan (1); Turkey (1); Hong Kong (1); Switzerland (1); Netherlands (1); Austria (1)	psychoeducation combined with cognitive behavioral therapy, group discussion element (photovoice and coming out proud), social skills training element, narrative enhancement or cognitive therapy elements	no active treatment; usual treatment	10-40 sessions	**Psychoeducation versus usual treatment:**<br><br>**- changes in internalized stigma of mental illness** (SMD= -0.40, 95% CI -0.64 to -0.16, I^2= 17%, p= 0.001)<br><br>**Self-stigma reduction program (photovoice, narrative enhancement/cognitive therapy, recovery oriented) versus usual treatment:**<br><br>**- reduction in total internalized stigma of mental illness total score** (SMD= -0.43, 95% CI -0.72 to -0.14, I^2= 22%, p= 0.003)	Critically Low
Wood, 2016	randomised controlled trials (7), controlled trials (2) and cohort studies (3)	714	USA (4); UK (2); Canada (1); Hong Kong (1); Switzerland (1); Portugal (1); Japan (1); Israel (1)	Psychosocial interventions (including cognitive behavior therapy, psychoeducation and social skills training)	Standard care or usual care, Waiting list control or Newspaper Reading group	The average number of sessions offered by the RCTs was 12.71 sessions (range 3–20), and 11.4 (range 6–20) by other studies.<br><br>The majority of studies utilized a group format intervention and only one study offered individual therapy	**- improvement in internalized stigma at the end of the therapy** was not significant = (Hedges' g 0.24, 95% CI -0.06 to 0.53, p=0.11)<br><br>**-improvement in internalized stigma at follow up (3 weeks to 4 months)** was not significant (Hedges' g 0.21, 95% CI −0.08 to 0.50, p = 0.16)	Low

Xu, 2017	randomised controlled trials (15), controlled trials (2)	2,373	China (16); Hong Kong (1)	Psychoeducation + usual psychiatric care or Cognitive Behavioral Therapy + usual psychiatric care	usual psychiatric care	4 weeks – 1 year or from 5 – 24 sessions	**Psychoeducation or Cognitive Behavioral Therapy versus usual psychiatric care:**<br><br>**-effects on perceived/experienced/ anticipated stigma** (SMD: 0.84, 95% CI 0.54 to 1.14, I^2= 87%, p< 0.001)<br><br>**-effects on self-prejudice** (SMD: 0.72, 95% CI: 0.51 to 0.93; I^2= 51%, p< 0.01)<br><br>**- effects on stigma coping** (SMD: 0.86, 95% CI: 0.60 to 1.15, I^2= 74%, p< 0.01)<br><br>**-improve on quality of life** (SMD: 0.75, 95% CI: 0.23 to 1.26; I^2= 84%, p=0.004)<br><br>**-improve on depression symptoms** (SMD: 0.77, 95% CI: 0.25 to 1.30, I^2= 89%, p< 0.01)<br><br>**- improve on anxiety symptoms** (SMD: 0.57, 95% CI: 0.34 to 0.81; I^2= 29%, p< 0.01)<br><br>**Subgroup analysis:**<br>Cognitive Behavioral Therapy (k=6, SMD: 0.90, 95% CI 0.31 to 1.49) had a similar effect as psychoeducation (k=8, SMD: 0.80, 95% CI 0.46 to 1.41) on perceived/ experienced/ anticipated stigma (χ2 =0.08, p=0.77).<br><br>Psychoeducation (k=3, SMD: 0.82, 95% CI 0.67 to 0.96) was more effective than Cognitive Behavioral Therapy (k=1, SMD: 0.29, 95% CI −0.20 to 0.78) in reducing self-	Critically low

							prejudice ($\chi2$ =4.09, p=0.04).<br><br>Cognitive Behavioral Therapy (k=1, SMD: 2.47, 95% CI 1.80 to 3.14) was superior to psychoeducation (k=7, SMD: 0.72, 95% CI 0.60 to 0.83) in improving coping with stigma ($\chi2$=25.79, p< 0.01).	
Morgan, 2018	randomised controlled trials (62)	9,002	North America (32); Europe (22); Asia (4); Australia (3); South America (1)	contact interventions, educational interventions, mixed contact and education, family psychoeducation programs, and hallucination simulations	waitlist, no intervention, treatment as usual or attention control	Duration of contact varied 1 - 105 min, with a median of 15 min.<br>The any anti-stigmatising effects were not examined beyond eight weeks, with most follow-ups only one week after the intervention	**Post Intervention:**<br><br>**Contact interventions**:<br>**-reductions in stigmatising attitudes:** (d=0.39, 95% CI: 0.22 to 0.55) and **desire for social distance** (d=0.59, 95% CI: 0.37 to 0.80)<br><br>**Education interventions:**<br>**-reductions in stigmatising attitudes** (d=0.30, 95% CI 0.14 to 0.47) **and desire for social distance** (d=0.27, 95% CI 0.08 to 0.46)<br><br>**Mixed contact & education interventions:**<br><br>**-reductions in stigmatising attitudes** (d= 0.32, 95% CI 0.08 to 0.56) **and desire for social distance** (d=0.43, 95% CI 0.01 to 0.86)<br><br>- **Family psychoeducation**: - **reductions in stigma post-intervention** (d=0.41, 95% CI 0.11 to 0.70).<br><br>**Follow up ≤ 6 months:**<br>-effects were not significant in any type of intervention.	Low

Table S.1 (continued): Included reviews (for strategy 3 – Intensive case management).

Author, year	Type and number of primary studies	Number of participants	Countries (studies)	Intervention	Comparator	Type of outcomes measure	Primary outcomes	AMSTAR 2
	**STRATEGY 3: INTENSIVE CASE MANAGEMENT**							
Burns, 2007	randomised controlled trials (29)	1,996	not reported	intensive case management (caseload up to and including 20)	standard care (from a community mental health team or outpatient clinic) or low intensity case management (caseload greater than 20) in people with severe mental disorder living in the community	not reported	**-hospital use at baseline** (coefficient −0.23, 95%CI−0.36 to −0.09, p=0.001); **-hospital use in control groups** (coefficient −0.44, CI 95% −0.57 to −0.31);	Low
Dieterich, 2017	randomised controlled trials (40)	7,524	Australia, Canada and USA (27); Europe (12); China (1);	Intensive case management (package of care shaped on the Assertive Community Treatment model, Assertive Outreach model or Case Management model; with a caseload up to 20 people)	**non-intensive case** (package of care shaped on the Assertive Community Treatment model, Assertive Outreach model or Case Management model; with over 20 people) or standard care community or outpatient model of care not specifically shaped on either the model of Assertive Community Treatment and Case Management, and	short term (up to 6 months), medium term (7-12months), and long term (over 12 months).	**Intensive case management versus standard care:**<br><br>**-reduced mean of the number of days in hospital per month** (MD: -0.86, 95% CI -1.37 to -0.34);<br><br>**-outcome global state** (RR: 0.68, 95% CI 0.58 to 0.79)<br><br>**-reducing death by suicide** (RR: 0.68, 95% CI 0.31 to 1.51)<br><br>**-social functioning the effect on unemployment** (RR: 0.70, 95% CI 0.49 to 1.0);<br><br>**-participant satisfaction** by short term (RR:6.20, 95% CI 2.60 to 9.80); by	High

					not working within a designated named package or approach to care)		medium term (RR: 1.93, 95% IC 0.86 to 3.01, I^2=0%); and by long term (RR: 3.23, 95% CI 2.31 to 4.14; I^2=0%);<br><br>**Intensive case management versus no standard care:**<br><br>**-reduced mean of the number of days in hospital per month** (MD: -0.08, 95% CI -0.37 to 0.21);<br><br>**-reducing death by suicide** (RR: 0.88, 95% CI 0.27 to 2.84);<br><br>**-social functioning the effect on unemployment** (RR: 1.46, 95% CI 0.45 to 4.74)	

Table S.1 (continued): Included reviews (for strategy 4 – Community mental health teams).

Author, year	Type and number of primary studies	Number of participants	Countries (studies)	Intervention	Comparator	Type of outcomes measure	Primary outcomes	AMSTAR 2
	**STRATEGY 4 - COMMUNITY MENTAL HEALTH TEAMS**							
Malone, 2007	randomised controlled trials (3)	587	UK (3)	management of care from community mental health team	Standard or usual care (normal care in the area concerned, non-team community care)	3- 12 months	-**death** by suicide and in suspicious circumstances (RR: 0.49, 95% CI 0.1 to 2.2; I^2= 0%)<br><br>-**leaving study early or up to 12 months** (RR: 1.10, 95% CI 0.68 to 1.78, I^2= 0%)<br><br>-**hospital admission** (RR: 0.81, 95% CI 0.67 to 0.97, I^2= 28%)<br><br>-**satisfaction with the service** (RR:0.37, 95% CI 0.18 to 0.79)<br><br>-**service use -use of Accident and emergency and general hospital up to 12 months:** (RR: 0.86, 95% CI 0.66 to 1.12, I^2= 44%)<br><br>-**service use – contact with primary care up to 12 months** (RR: 0.94, 95% CI 0.80 to 1.11, I^2= 0%)<br><br>-**service use – contact with social services up to 12 months** (RR: 0.76, 95% CI 0.58 to 1.01, I^2= 0%)<br><br>-**social functioning– police contacts** (RR: 2.07, 95% CI 1.08 to 3.97, I^2=53%)	Moderate

Table S.1 (continued): Included reviews (for strategy 5 – Assisted living).

Author, year	Type and number of primary studies	Number of participants	Countries (studies)	Intervention	Comparator	Type of outcome Measure	Primary outcomes	AMSTAR 2
	**STRATEGY 5 – ASSISTED LIVING**							
Leff, 2009	randomised controlled trials (6); other design not reported (38)	13,436	not reported	model housing (residential care and treatment housing; supported housing interventions; permanent Supported housing)	Non-model housing	6 months to 5 years	**Residence care and treatment versus non-model housing:**<br><br>**-housing stability:** (effect size= 0.48, p< 0.05)<br><br>**-reduction in psychiatric symptoms:** (effect size= 0.65, p<0.05)<br><br>**-reduction in hospitalization:** (effect size= 0.34, p< 0.05)<br><br>**-reduction in alcohol abuse:** (effect size= 0.87, p> 0.05)<br><br>**-reduction in drug abuse:** (effect size= 0.41, p> 0.05)<br><br>**-increased employment:** (effect size= 0.27, p> 0.05)<br><br>**-increased satisfaction:** (effect size= 0.07, p> 0.05)<br><br>**Residential continuum versus non-model housing:**<br><br>**-housing stability:** (effect size= 0.80, p< 0.05)<br><br>**-reduction in psychiatric**	Low

							**symptoms:** (effect size= 0.68, p> 0.05)<br><br>**-reduction in alcohol abuse:** (effect size=0.07, p> 0.05)<br><br>**-reduction in drug abuse:** (effect size=0.3, p> 0.05)<br><br>**-increased satisfaction:** (effect size=0.55, p> 0.05)<br><br>**Permanent Supported Housing versus non-model housing:**<br><br>**-housing stability:** (effect size=0.63, p< 0.05)<br><br>**-reduction in psychiatric symptoms:** (effect size= 0.08, p> 0.05)<br><br>**-reduction in hospitalization:** (effect size= 0.72, p< 0.05)<br><br>**-reduction in alcohol abuse:** (effect size= 0.21, p> 0.05)<br><br>**-reduction in drug abuse:** (effect size= 0.51, p> 0.05)<br><br>**-increased employment:** (effect size= 0.27, p> 0.05)<br><br>**-increased satisfaction:** (0.73, p< 0.001)<br><br>**Non-model housing:**<br><br>**-housing stability:** (effect size=	

							-0.63, p> 0.05)<br><br>**-reduction in psychiatric symptoms:** (effect size= -0.11, p> 0.05)<br><br>**-reduction in hospitalization:** (effect size= -0.33, p> 0.05)<br><br>**-reduction in alcohol abuse:** (effect size= 0.06, p> 0.05)<br><br>**-reduction in drug abuse:** (effect size= 0.2, p> 0.05)<br><br>**-increased satisfaction:** (effect size= -0.38, p> 0.05)	
McPherson, 2018	Total in “deinstitutionalization” subgroup = 28: cohort studies (24), quasi-experimental (2), single case control (1), randomized controlled trial (1)<br><br>Total in review= 115	6,516<br><br>(but one of study did not declare the total number of participants)	not reported separately	mental health supported accommodation (defined as any service that provided support, delivered predominately by non-professionally qualified staff, to people with mental health problems living in community-based accommodation, either aloneor in shared settings)	none or accommodation settings (at home with family or friend, in own house), other type of model accommodation	6 months to 13 years of follow up	Due to the heterogeneity of the retrieved studies, in terms of the design of the study, type of supported housing, population, and outcomes, the data were unfeasible to summarized. Synthesis narrative of high and moderate quality studies suggested a trend toward improvement in symptoms, social functioning, stability and in a reduction the rate of hospitalization	Low

Table A.1 (continued): Included reviews (for strategy 6 – Interventions for acute psychiatric episodes).

Author, year	Type and number of primary studies	Number of participants	Countries (studies)	Intervention	Comparator	Type of outcome measure	Primary outcomes	AMSTAR 2
	**STRATEGY 6 - INTERVENTIONS FOR ACUTE PSYCHIATRIC EPISODES**							
Murphy, 2015	randomised controlled trials (8)	1,144	Australia (1); Canada (2); USA (2); UK (3)	crisis intervention (any type of crisis-orientated treatment of an acute psychiatric episode by staff with a specific remit to deal with such situations, in and beyond ’office hours’)	standard care (normal care given to those suffering from acute psychiatric episodes in the area concerned)	3 months – 2 years	**-reduction of repeat admissions to hospital at six months** (RR: 0.75, 95% CI 0.50 to 1.13; I^2= 80%);<br><br>**-improve mental state according to Brief Psychiatric Rating Scale, three months** (MD: -4.03, 95% CI -0.18 to 0.12);<br><br>**-improve global state according to Global Assessment Scale, 20 months** (MD: 5.70, 95% CI -0.26 to 11.66)<br><br>**-satisfaction with the care, 20 months crisis according to Client Satisfaction Questionnaire** (MD: 5.40, 95% CI 3.91 to 6.89)<br><br>**-reduction of family burden at six months** (RR: 0.34, 95% CI 0.20 to 0.59)<br><br>**-quality of life scores at six months according to Manchester Short Assessment of quality of life** (MD: -1.50, 95% CI -5.15 to 2.15)	High
Wheeler, 2015	Total studies (69), which 21 were used in quantitative	14,833 (but, seven studies did not report	Australia (3), Germany (1), USA (2), UK (15)	crisis resolution teams	usual treatment; other crisis resolution teams model	not reported	The quantitative synthesis was not feasible due to different designs of the retrieved studies such as type of studies, type of outcomes and	Low

	analysis	total n)					settings. However, narrative synthesis suggests that crisis resolution teams reduces hospital admissions and recommend as key characteristics: 24 hours service provision, presence of a psychiatrist in the team, communication and integration with other local mental health services, high quality of training.	
