# Supplementary material for: Improving Care for Deinstitutionalized People With Mental Disorders: Experiences of the Use of Knowledge Translation Tools
Source: Front Psychiatry. 2021 Apr 26;12:575108. doi: 10.3389/fpsyt.2021.575108 (PMC8109270; doi:10.3389/fpsyt.2021.575108)
Supplement: Supplementary file 2 [file Table_2.PDF]

SUPPLEMENTARY MATERIAL

Table S2 - Excluded studies

Table S.2: Excluded studies.

Author, date	Reason for exclusion
**Akiyama, 2008**	Not a systematic review
**Arboleda-Fiórez, 1998**	Not a systematic review
**Ashton, 2018**	Not a systematic review
**Appathurai, 1986**	Not a systematic review
**Aubry, 2015**	Not a systematic review
**Ayano, 2018**	Not an outpatient care strategy or follow-up for deinstitutionalized patients
**Baptista, 2011**	Focus only on patients with bipolar disorders
**Bemak, 1985**	Not a systematic review
**Campbell, 2009**	Not an outpatient care strategy or follow-up for deinstitutionalized patients
**Catty, 2002**	Included in another more recent systematic review
**Chan, 2009**	Not a systematic review
**Chilvers, 2010**	Not an outpatient care strategy or follow-up for deinstitutionalized patients
**Collard, 2014**	Not a systematic review
**Dixon, 1995**	Not a systematic review
**Hazel, 2017**	Not a systematic review
**Hailemariam, 2016**	Not an outpatient care strategy or follow-up for deinstitutionalized patients
**Henderson, 1998**	Not a systematic review
**Kanapp, 2011**	Not comparison among strategies for outpatient follow-up and care
**Kohrt, 2018**	Not an outpatient care strategy or follow-up for deinstitutionalized patients
**Kozma, 2009**	Focus only on adults with intellectual disorders
**Kronenberg, 2017**	Not an outpatient care strategy or follow-up for deinstitutionalized patients
**Kunitoh, 2013**	Not a systematic review
**Kyle,2008**	Not an outpatient care strategy or follow-up for deinstitutionalized patients
**Ly, 2015**	Not a systematic review
**Lyman, 2014**	Not a systematic review
**Marshall,2011**	Not an outpatient care strategy or follow-up for deinstitutionalized patients
**Mascayano, 2016**	Not an outpatient care strategy or follow-up for deinstitutionalized patients
**Mccrone, 2009**	Not a systematic review
**Mittal, 2012**	Not a systematic review
**O'Campo, 2009**	Not an outpatient care strategy or follow-up for deinstitutionalized patients
**Peterson, 2013**	Not an outpatient care strategy or follow-up for deinstitutionalized patients
**Petrello, 2017**	Not a systematic review
**Petretto, 2013**	Not a systematic review

**Ramonet, 2013**	Not a systematic review
**Ran, 2003**	Not a systematic review
**Reily, 2013**	Not an outpatient care strategy or follow-up for deinstitutionalized patients
**Roy,2014**	Not a systematic review
**Ryu, 2006**	Not a systematic review
**Sampaio, 2006**	Not a systematic review
**Shah, 2014**	Focus only on patients with stress
**Simmonds, 2001**	Included in another more recent systematic review
**Tursi, 2013**	Focus only on patients with depression
**Vazquez-Bourgon, 2012**	Not a systematic review
**Wash, 2010**	Focus only on adults with intellectual disorders
**White, 2018**	Not an outpatient care strategy or follow-up for deinstitutionalized patients (systematic review protocol)
**Winters, 2015**	Not a systematic review
**Winkler, 2016**	Not an outpatient care strategy or follow-up for deinstitutionalized patients
**Worral, 2018**	Not a systematic review
**WHO, 2014**	Not a systematic review
**Ziguras, 2000**	Not an outpatient care strategy or follow-up for deinstitutionalized patients
