# Supplementary material for: Improving Care for Deinstitutionalized People With Mental Disorders: Experiences of the Use of Knowledge Translation Tools
Source: Front Psychiatry. 2021 Apr 26;12:575108. doi: 10.3389/fpsyt.2021.575108 (PMC8109270; doi:10.3389/fpsyt.2021.575108)
Supplement: Supplementary file 3 [file Table_3.PDF]

SUPPLEMENTARY MATERIAL
Table S3 – AMSTAR results.

Table S.3: Critical appraisal of studies included using the AMSTAR 2 tool.

Author, year	Q1	Q2	Q3	Q4	Q5	Q6	Q7	Q8	Q9	Q10	Q11	Q12	Q13	Q14	Q15	Q16	Ranking of quality[&]
**STRATEGY 1: PSICOEDUCAÇÃO**																	
Pilling 2002	Y	NA**	N	N	Y	Y	N	Y	N	N	Y	N	N	Y	NA*	N	Critically low
Lincoln 2007	Y	NA**	N	PY	Y	Y	N	Y	PY	N	Y	Y	Y	Y	Y	Y	Low
Xia2011	Y	Y	N	Y	Y	Y	Y	Y	Y	N	Y	Y	Y	Y	Y	Y	Moderate
Zhao 2015	Y	Y	N	Y	Y	Y	Y	Y	Y	Y	Y	Y	Y	Y	NA*	Y	High
**STRATEGY 2: ANTI-STIGMA PROGRAMS**																	
Wood 2016	Y	Y	N	PY	N	N	Y	Y	Y	N	Y	N	N	Y	NA*	Y	Low
Xu 2017	Y	Y	N	PY	Y	Y	N	PY	Y	N	Y	N	N	Y	Y	N	Critically low
Tsang 2016	Y	N	N	PY	Y	Y	N	Y	Y	N	Y	Y	Y	N	NA*	N	Critically low
Morgan 2018	Y	Y	Y	PY	N	Y	N	Y	Y	N	Y	Y	Y	Y	Y	Y	Low
**STRATEGY 3: INTENSIVE CASE MANAGEMENT**																	
Burns 2007	Y	NA**	N	PY	Y	Y	N	N	Y	N	Y	Y	Y	Y	Y	Y	Low
Dieterich 2017	Y	Y	N	Y	Y	Y	Y	Y	Y	Y	Y	Y	Y	Y	Y	Y	High
**STRATEGY 4: COMMUNITY MENTAL HEALTH TEAMS**																	
Malone 2017	Y	Y	N	Y	Y	Y	Y	Y	Y	N	Y	Y	Y	Y	NA*	Y	Moderate
**STRATEGY 5: ASSISTED LIVING**																	
Leff 2009	Y	NA**	N	Y	Y	Y	PY	PY	Y	N	Y	Y	Y	N	N	Y	Low
McPherson 2018	Y	Y	N	PY	Y	Y	N	Y	Y	N	NA	NA	Y	Y	NA	Y	Low
**STRATEGY 6: INTERVENTIONS FOR ACUTE PSYCHIATRIC EPISODES**																	
Murphy 2015	Y	Y	N	Y	Y	Y	Y	Y	Y	Y	Y	Y	Y	Y	NA*	Y	High
Wheeler2015	Y	Y	Y	Y	Y	Y	N	PY	Y	N	NA	NA	Y	Y	NA	Y	Low

Y, yes; PY, partial yes; N, no; N/A: not applicable; N/A*: not applicable, because there were 10 or fewer studies per outcome; NA**: the systematic review protocol records base PROSPERO was available virtually only in February 2011; ** PROSPERO started on line registration in 2011.

[&]High: no or one non-critical weakness; Moderate: more than one non-critical weakness; Low: one critical flaw with or without non-critical weaknesses; Critically low: more than one critical flaw with or without non-critical weaknesses

DOMAINS IN AMSTAR 2:

Q1: Did the research questions and inclusion criteria for the review include the components of PICO?

Q2 (critical domain): Did the report of the review contain an explicit statement that the review methods were established prior to the conduct of the review and did the report justify any significant deviations from the protocol?

Q3: Did the review authors explain their selection of the study designs for inclusion in the review?

Q4 (critical domain): Did the review authors use a comprehensive literature search strategy?

Q5: Did the review authors perform study selection in duplicate?

Q6: Did the review authors perform data extraction in duplicate?

Q7 (critical domain): Did the review authors provide a list of excluded studies and justify the exclusions?

Q8: Did the review authors describe the included studies in adequate detail?

Q9 (critical domain): Did the review authors use a satisfactory technique for assessing the risk of bias (RoB) in individual studies that were included in the review?

Q10: Did the review authors report on the sources of funding for the studies included in the review?

Q11 (critical domain): If meta-analysis was performed did the review authors use appropriate methods for statistical combination of results?

Q12: If meta-analysis was performed, did the review authors assess the potential impact of RoB in individual studies on the results of the meta-analysis or other evidence synthesis?

Q13 (critical domain): Did the review authors account for RoB in individual studies when interpreting/discussing the results of the review?

Q14: Did the review authors provide a satisfactory explanation for, and discussion of, any heterogeneity observed in the results of the review?

Q15 (critical domain): If they performed quantitative synthesis did the review authors carry out an adequate investigation of publication bias (small study bias) and discuss its likely impact on the results of the review?

Q16: Did the review authors report any potential sources of conflict of interest, including any funding they received for conducting the review?
