# Supplementary material for: Improving Care for Deinstitutionalized People With Mental Disorders: Experiences of the Use of Knowledge Translation Tools
Source: Front Psychiatry. 2021 Apr 26;12:575108. doi: 10.3389/fpsyt.2021.575108 (PMC8109270; doi:10.3389/fpsyt.2021.575108)
Supplement: Supplementary file 4 [file Table_4.PDF]

SUPPLEMENTARY MATERIAL

Table S4 - Common barriers in mental health and possible solutions.

Table S.4: Common barriers in mental health and possible solutions.

Barriers	Possible solutions to barriers
**Health care professionals**	
-Professional resistance: rigid attitudes of professionals and reluctance to make any changes in their work (Shen et al., 2017).<br>-Scarcity of qualified and credentialed mental health workers (Kakuma et al., 2011;Shen et al., 2017).<br>- Deficiency of trained mental health team and poorly equipped staffed health care(Kakuma et al., 2011).<br>-Ineffectiveness of multidisciplinary teams due to inconsistent practices or practices that are not documented clearly, poor communication, and lack of leadership (Woody et al., 2018).	-Strengthening institutional capacity to implement effective training programmes and continuous education (Kakuma et al., 2011).<br>-Development of a new and wide range of cadres, delegating and shifting tasks within and across sectors (Kakuma et al., 2011).<br>-Good communication within teams, defined leadership, and clear documentation practices (Woody et al., 2018).
**Government officials**	
-Lack of political priority and budgetary support for mental health (Shen et al., 2017).<br>-Poor leadership and underqualified managers (Shen et al., 2017).<br>-Enactment of mental health policies without real changes in practice (WHO, 2014).	-Need for political leaders with will, commitment and ownership (Shen et al., 2017).<br>-Cooperation from all levels of government to develop, implement and review mental health policy and legislation (Bhugra et al., 2018).
**Patients**	
-Social stigma, discrimination, cultural beliefs, religious roots and negative societal responses (Stuart, 2016).<br>-Self-stigma and isolation, (Tsang et al., 2016;Morgan et al., 2018).<br>-Lack of public education (WHO, 2001).<br>-Difficulties in entering the formal labour market (Kinoshita et al., 2013) and becoming involved in other activities.	-Public awareness and education campaigns focusing on the frequency of mental disorders, their treatment, recovery and the human rights of people with mental disorders (WHO, 2001).<br>-Supported employment (Kinoshita et al., 2013).<br>-Provision of different modes of occupation to give a sense of purpose to life (Burgoyne, 2014).<br>-Peer support services (Walker and Bryant, 2013;Chinman M., 2014).
**Others (Lack of partnership with other sectors and knowledge translation)**	
-Lack of partnership formation with other sectors (Shen et al., 2017).<br>-Knowledge–action gap: failure to incorporate science into routine healthcare practice and health policy (Shidhaye, 2015;Chinman et al., 2017).<br>-Gap in indicators for mental health and the available information system, particularly in low- and middle-income countries. No or little data are available about mental health service needs, coverage, quality and	-Configuration of proactive partnerships with clearly defined roles and that pursue evidence-based innovations (WHO, 2001): i) partnerships between governments and non-governmental organizations and private practitioners (Shen et al., 2017); ii) partnerships among universities, agencies, and local and international organizations (WHO, 2001;Kakuma et al., 2011).<br>-Adoption of scientific implementation frameworks to advance equity and decrease disparities (Chinman et al.,

resource demands (Upadhaya et al., 2016;Ahuja et al., 2018).	2017); this could be facilitated by an external team (Shidhaye, 2015).<br><br>-Development and inclusion of standard indicators for mental health and a reporting system to monitor the coverage and quality of services(Ahuja et al., 2018).

REFERENCES

Ahuja, S., Shidhaye, R., Semrau, M., Thornicroft, G., and Jordans, M. (2018). Mental health information systems in resource-challenged countries: experiences from India. *BJPsych Int* 15**,** 43-46. doi:10.1192/bji.2017.6

Bhugra, D., Pathare, S., Joshi, R., Kalra, G., Torales, J., and Ventriglio, A. (2018). A review of mental health policies from Commonwealth countries. *Int J Soc Psychiatry* 64**,** 3-8. doi:10.1177/0020764017745108

Burgoyne, J. (2014). Mental health and the settings of housing support – a systematic review and conceptual model. *Housing, Care and Support* 17**,** 26-40. doi:https://doi.org/10.1108/HCS-10-2013-0018

Chinman, M., Woodward, E.N., Curran, G.M., and Hausmann, L.R.M. (2017). Harnessing Implementation Science to Increase the Impact of Health Equity Research. *Med Care* 55 Suppl 9 Suppl 2**,** S16-s23. doi:10.1097/mlr.0000000000000769

Chinman M., G.P., Dougherty R.H. Et Al. (2014). Peer Support Services for Individuals With Serious Mental Illnesses: Assessing the Evidence. P*sychiatric Services* 65 (4), 429-41. doi: 10.1176/appi.ps.201300244

Kakuma, R., Minas, H., Van Ginneken, N., Dal Poz, M.R., Desiraju, K., Morris, J.E., Saxena, S., and Scheffler, R.M. (2011). Human resources for mental health care: current situation and strategies for action. *Lancet* 378**,** 1654-1663. doi:10.1016/s01406736(11)61093-3

Kinoshita, Y., Furukawa, T.A., Kinoshita, K., Honyashiki, M., Omori, I.M., Marshall, M., Bond, G.R., Huxley, P., Amano, N., and Kingdon, D. (2013). Supported employment for adults with severe mental illness. *Cochrane Database Syst Rev***,** Cd008297. doi:10.1002/14651858.CD008297.pub2

Morgan, A.J., Reavley, N.J., Ross, A., Too, L.S., and Jorm, A.F. (2018). Interventions to reduce stigma towards people with severe mental illness: Systematic review and meta-analysis. *J Psychiatr Res* 103**,** 120-133. doi:10.1016/j.jpsychires.2018.05.017

Shen, G.C., Eaton, J., and Snowden, L.R. (2017). Mainstreaming Mental Health Care in 42 Countries. *Health Syst Reform* 3**,** 313-324. doi:10.1080/23288604.2017.1356424

Shidhaye, R. (2015). Implementation Science for closing the treatment gap for mental disorders by translating evidence base into practice: experiences from the PRIME project. *Australas Psychiatry* 23**,** 35-37. doi:10.1177/1039856215609771

Stuart, H. (2016). Reducing the stigma of mental illness. *Global Mental Health (Cambridge, England)* 3. doi:doi:10.1017/gmh.2016.11

Tsang, H.W., Ching, S.C., Tang, K.H., Lam, H.T., Law, P.Y., and Wan, C.N. (2016). Therapeutic intervention for internalized stigma of severe mental illness: A systematic review and meta-analysis. *Schizophr Res* 173**,** 45-53. doi:10.1016/j.schres.2016.02.013

Upadhaya, N., Jordans, M.J.D., Abdulmalik, J., Ahuja, S., Alem, A., Hanlon, C., Kigozi, F., Kizza, D., Lund, C., Semrau, M., Shidhaye, R., Thornicroft, G., Komproe, I.H., and Gureje, O. (2016). Information systems for mental health in six low and middle income countries: cross country situation analysis. *Int J Ment Health Syst* 10**,** 60. doi:10.1186/s13033-016-0094-2

Walker, G., and Bryant, W. (2013). Peer support in adult mental health services: a metasynthesis of qualitative findings. *Psychiatr Rehabil J* 36**,** 28-34. doi:10.1037/h0094744

WHO (2001). World Health Organization. World Health Report 2001. Mental health: new understanding, new hope. WHO: Geneva, Switzerland; 2001.

WHO (2014). World Health Organization and the Gulbenkian Global Mental Health Plataform. Innovation in deinstitutionalization: a WHO expert survey. Geneva: World Health Organization, 2014.

Woody, C.A., Baxter, A.J., Harris, M.G., Siskind, D.J., and Whiteford, H.A. (2018). Identifying characteristics and practices of multidisciplinary team reviews for patients with severe mental illness: a systematic review. *Australas Psychiatry* 26**,** 267-275. doi:10.1177/1039856217751783
