# Supplementary material for: Improving Care for Deinstitutionalized People With Mental Disorders: Experiences of the Use of Knowledge Translation Tools
Source: Front Psychiatry. 2021 Apr 26;12:575108. doi: 10.3389/fpsyt.2021.575108 (PMC8109270; doi:10.3389/fpsyt.2021.575108)
Supplement: Supplementary file 5 [file Data_Sheet_1.PDF]

Supplementary Materials
Data Sheet 1 - Search strategy

1. Community Mental Health Services.mp. or exp Community Mental Health Services/
2. Case Management.mp. or exp Case Management/
3. Managed Care Programs.mp. or exp Managed Care Programs/
4. Community Mental Health Centers.mp. or exp Organizational Case Studies/ or Patient Care Team/
5. Community Mental Health Centers.mp. or exp Community Mental Health Centers/
6. Psychoeducation
7. Community mental health team
8. Crisis intervention
9. Housing support
10. Mental Disorders.mp. or exp Mental Disorders/
11. deinstitutionalization.mp. or exp Deinstitutionalization/
12. 1 or 2 or 3 or 4 or 5 or 6 or 7 or 8 or 9
13. 10 and 12
14. 11 and 13
15. limit 14 to “review articles”
